# 

**Published:** 1889-08-31

**Authors:** 


### MISS FLORENCE NIGHTINGALE.

### SPECIAL ANNOUNCEMENT.

The portrait of Miss Nightingale is now completed, and will be published in the September Monthly Part of The Hospital; it having been reproduced at great cost and perfection. Every member of the nursing profession will now be able to acquire the likeness of one, who, though raised to pre-eminence among women by a life of self-sacrifice and philanthropic labour, has nevertheless shrunk from the publicity which fame always brings.

The Monthly Number will be issued at 6d., as usual, but orders should be sent in early, as the demand is expected to be very great. Copies of the portrait alone are obtainable at 3d. each. A biographical sketch of Miss Nightingale appeared in The Hospital of July 27th.

### NOTICE TO CORRESPONDENTS.

All MS., Letters, Books for Review, and other matters intended for the Editor, should be addressed The Editor, The Lodge, Porchester Square, London, W.

### Notice to Advertisers and Subscribers.

All Advertisements, Orders for Copies of The Hospital, and Business Communications should be addressed to The Publisher, *not to the Editor*, The Hospital, 139-140, Salisbury-court, Fleet-street, E.C.

Vol. V., now ready, 4s., post free. Cases for binding, may be had o the Publisher, at 1s. 6d. each.

### To Residents, Secretaries, and Matrons.

The Editor will be glad to have the Regulations and each Report a issued by Asylums, Hospitals, Convalescent and Nursing Institutions, a well as those of other Charities, and an early copy in duplicate of al Appeals and Festival Notices, etc., addressed to *The Editor, The Lodge, Porchester-square, London, W.* Any superintendent, secretary, matron, or other chief official who regularly sends such information may be registered, on application to the Publisher, to receive free of cost a cover or binding The Hospital in half-yearly volumes.

### Amusements and Relaxation.

### Second Quarterly Word Competition Commenced July 6th, ends September 28th.

Three Prizes of 15s., 10s., 5s., will be given for the largest number of words derived from the words set for dissection.

Proper names, abbreviations, foreign words, words of less than four letters, and repetitions are barred; plurals, and past and present participles of verbs, are allowed. Nuttall's *Standard* dictionary only to be used.

N.B.—Word dissections must be sent in WEEKLY, arranged alphabetically, with correct total affixed.

The word for dissection for this, the *Ninth* week of the quarter, being

"C O R W E N."

**Results of Seventh Week.**

| Names. | Aug. 22nd. | Totals. | Names. | Aug. 22nd. | Totals. |
|---|---|---|---|---|---|
| Nurse Duty | 44 | 460 | Leirion | — | 195 |
| N'importe Qui | 42 | 421 | Matron | — | 174 |
| Jumps | — | 30 | F. C. J. | 33 | 305 |
| Tinie | — | 186 | Percy Verance | — | 18 |
| Patience | 44 | 466 | Gleniffer | — | 175 |
| Amicus | 36 | 430 | Gypsye | 40 | 401 |
| Broxbourne | — | 398 | Ladyr | — | 127 |
| Speedwell | 38 | 419 | A. B. | — | 170 |
| Sister | 43 | 444 | Essie | 35 | 361 |
| Lightowlers | 43 | 335 | W. G. R. | 34 | 356 |
| Lauriston | 36 | 324 | Cowboy Bill | — | 179 |
| Rattler | — | 28 | M. L. A. | — | 117 |
| Paignton | — | 334 | Little Tuppy | — | 110 |
| Fassifern | — | 141 | Embryo | — | 56 |
| W. R. E. | 43 | 448 | K. Jarman | — | 43 |
| Esperance | 42 | 460 | Reveal | — | 196 |
| M. W. | 42 | 465 | Jenny Wren | 36 | 353 |
| Isabel | 44 | 433 | Condorcet | 40 | 386 |
| Buxton | — | 167 | Electrician | 41 | 225 |
| Nux Vom | — | 263 | Pearl | — | 132 |
| Qu'appelle | 40 | 399 | R. W. | — | 43 |
| Spes | — | 24 | Nurse Amie | — | 89 |
| Secnarf | — | 229 | Sunshine | 36 | 143 |
| Rover | 41 | 206 | Juvenis | 23 | 23 |

**Conditions.**

I. Every paper sent in must be clearly written, and one side only must be used. All competitors must mark at the head of their papers the number of their competition.

II. Each paper must be signed by the author with his or her real name and address. A *nom de plume* may be added if the writer does not desire to be referred to by us by his real name. In the case of all prize-winners, however, the real name and address will be published.

III. All communications relating to prize competitions should be addressed to "The Prize Editor, The Hospital, 139 and 140, Salisbury-court, London, E.C." Competitors are requested not to include in letters, etc., to the Prize Editor communications on matters of other business. All letters must be fully prepaid.

IV. The Prize Editor of The Hospital is the sole judge of the competitions, and his decision is in all cases final and without appeal.

V. The Prize Editor reserves the right to publish any paper sent in, whether it receives a prize or not. He does not hold himself responsible for any MSS. sent, nor can he undertake to return rejected competitions.

### Scraps and Gleanings.

Hull Royal Infirmary appeals for £6,000.

Chicago employs five women as sanitary police.

Scarlet fever is on the increase again in London.

Enteric fever is to be isolated in future at Manchester.

The death is announced of Dr. Habershon, of Brook-street.

Diphtheria claimed over thirty victims in London alone last week.

The committee of Harrogate Bath Hospital issue a special appeal for funds.

Miss S. Gray has been appointed clinical assistant to Homerton Fever Hospital.

The death is announced of Dr. Chevallier, superintendent of Ipswich Borough Asylum.

Hull will keep Hospital Sunday on October 27th. Efforts will be made to raise £1,000.

Official sanction has been given to Mr. Baldwin Latham's scheme for the sewerage of Margate.

Bexley cannot raise £2,000 to build a cottage hospital. Generosity must be at a premium there.

At Exeter Hospital Saturday meeting two complaints against the Eye Infirmary were brought forward.

St. John's Convalescent Home at Cowleigh, which was opened in January last, is doing good work.

Weedon Hospital Sunday collection only amounted to £3 15s. It ought to reach at least five guineas.

Monkwearmouth Dispensary will in future be known as the Monkwearmouth and Southwick Hospital.

An Australian leper colony has been formed in Torres Straits, and appropriately named "Damien Island."

Madame Heine founded a dispensary for women and children at Paris. Last week her patients feted her.

Epsom railway employes have presented a handsome case of surgica instruments to the new cottage hospital.

The Home for Crippled Children now being built at Cheetham Hill, near Manchester, approaches completion.

The late Miss H. B. Denton has left £110 to Bootle Borough Hospital, and £1,100 to Liverpool Seamen's Orphanage.

Pontefract Dispensary acknowledges, with thanks, a cheque for £66 from the Friendly Societies' Sunday gathering.

The Dowager-Empress Augusta has given two candlesticks and a crucifix to the chapel of the Military Hospital, Hanover.

Clevedon Dispensary issues a forty-fifth annual report. Increasing numbers avail themselves of the benefits of this dispensary.

The Mayoress of Portsmouth tried to persuade ladies to collect on Hospital Saturday, but they one and all began to make excuse.

The West Ham Hospital is now all but completed, and arrangements are being made for the furnishing. The building has cost £6,710.

An inmate of an infirmary in Belgium went mad last Friday, and seizing a razor wounded twenty-five persons, three of them fatally.

Mons. Alexandre Jacques, who undertook to fast thirty days in Edinburgh, completed his task successfully on Saturday afternoon.

Leprosy still excites public interest; both *Blackwood's* and *Chamber's* provide realistic articles on this subject in their September numbers.

Dr. George Watt reports that large quantities of podophyllin are attainable in the Himalayas. This drug is valuable in diseases of the liver.

A friend of Dr. Humphrey's, of Limerick, has had the mistaken kindness to write a letter to the papers describing a successful operation performed by the doctor.

An anonymous donor has given £10,000 towards the establishment of the new bishopric in South Wales, conditional on the headquarters of the new see being at Swansea.

Dr. Wyss, of Geneva, speaks very highly of the ethereal tincture of iron, or Bestucheff's tincture, as it is usually called on the Continent, in cases of chronic Bright's disease.

Barnstaple Infirmary treated 512 in-patients and 2,208 out-patients last year. The expenditure was unusually large, as the drainage had had to be improved.

Mr. Adam Geck, late of Chiswell-street, London, has left by his will £300 to the German Hospital, Dalston, and £100 to the City of London Hospital for Diseases of the Chest.

An election for a coroner for Mid-Antrim took place last week. There were two candidates, Mr. Caruth and Dr. McCaw, the former being successful by a large majority.

Mr. Gaubert, the chemist whose thumb was fearfully lacerated by a rabid dog in Sudbury last week, whilst attempting to poison the animal, started on Saturday for the Pasteur establishment in Paris.

At Rome, from the 15th to the 17th (inclusive) of October next, will assemble the second congress of the Italian Medical Association. Among its special features will be reports on "Malarial Infection."

The Committee of the Animals' Institute have awarded the institute medals to Messrs. Garstins and the "A 1" muzzle. The exhibits are now on view at the Animals' Institute, 9, Kinnerton-street, Belgravia.

Dr. Kunze, in his inaugural thesis for the M.D. degree of the University of Kiel, publishes as a contribution to the diseases caused by the inhalation of dust a series of examinations of lungs so affected.

Mr. T. W. Cory, surgeon, of Bournemouth, has published a third edition of a pamphlet in connection with sick diet and applications, and which gives some valuable information regarding the treatment of the sick.

On August 13th, delegates from the various friendly, benefit, and trade societies met at the Old Friends' Hall, Upper St. Martin's-lane, London, for the purpose of presenting £139 to the secretary of the Children's Hospital, Great Ormond-street, being the net result of a demonstration held on June 30th.

